# Correction: Sustainable visions: Unsupervised machine learning insights on global development goals

**DOI:** 10.1371/journal.pone.0324556

**Published:** 2025-05-15

**Authors:** Alberto García-Rodríguez, Matias Núñez, Miguel Robles Pérez, Tzipe Govezensky, Rafael A. Barrio, Carlos Gershenson, Kimmo K. Kaski, Julia Tagüeña

There are errors in the Author Contributions. The correct contributions are,

**Conceptualization:** Alberto García-Rodríguez, Matias Núñez, Rafael A. Barrio, Carlos Gershenson, Kimmo K. Kaski, Julia Tagüeña

**Data curation:** Alberto García-Rodríguez, Matias Núñez, Miguel Robles Pérez, Tzipe Govezensky,

**Formal analysis:** Matias Núñez, Miguel Robles Pérez, Tzipe Govezensky, Rafael A Barrio, Kimmo K Kaski, Julia Tagüeña,

**Investigation:** Alberto García-Rodríguez, Matias Núñez, Miguel Robles Pérez, Tzipe Govezensky, Julia Tagüeña

**Methodology:** Matias Núñez, Tzipe Govezensky, Carlos Gershenson, Kimmo K. Kaski, Julia Tagüeña

**Project administration:** Rafael A Barrio, Kimmo K Kaski, Julia Tagüeña

**Supervision:** Kimmo K. Kaski

**Validation:** Kimmo K. Kaski, Julia Tagüeña

**Visualization:** Alberto García-Rodríguez, Matias Núñez, Miguel Robles Pérez, Tzipe Govezensky

**Writing – original draft:** Alberto García-Rodríguez, Matias Núñez, Miguel Robles Pérez, Tzipe Govezensky, Rafael A Barrio, Kimmo K Kaski, Julia Tagüeña

**Writing – review & editing:** Alberto García-Rodríguez, Matias Núñez, Miguel Robles Pérez, Tzipe Govezensky, Rafael A Barrio, Carlos Gershenson, Kimmo K Kaski, Julia Tagüeña
