# Supplementary material for: Sustainable visions: unsupervised machine learning insights on global development goals
Source: PLoS One. 2025 Mar 12;20(3):e0317412. doi: 10.1371/journal.pone.0317412 (PMC11902278; doi:10.1371/journal.pone.0317412)
Supplement: S1 Text — (PDF) [file pone.0317412.s001.pdf]

Supplementary material

1 Definition of SDG

In here we show in a familiar graphical way the description of the SDG's to facilitate their identification if needed.

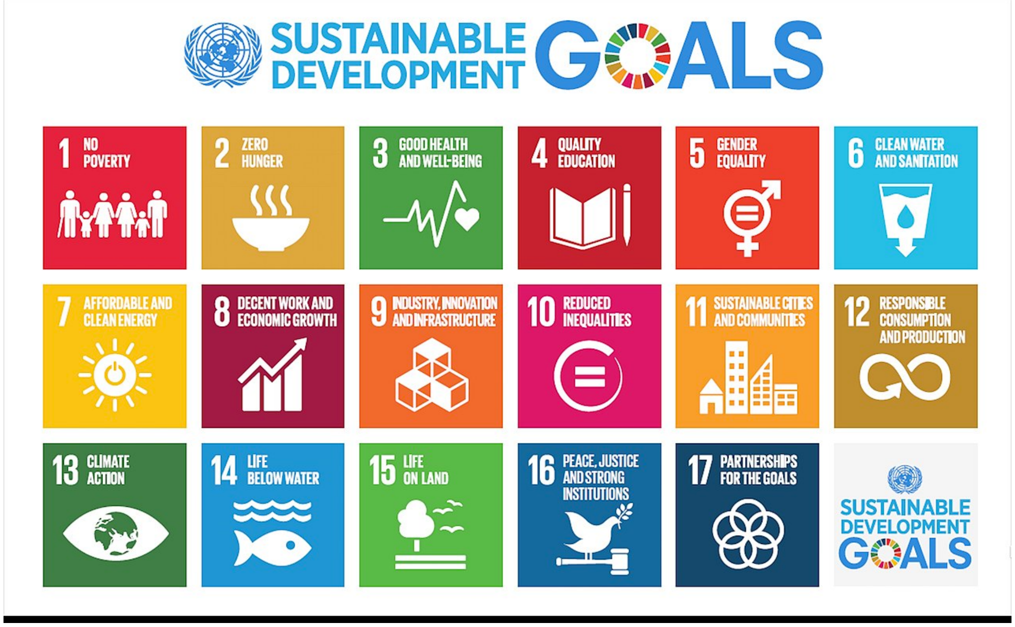

Fig 1.1. Meaning of the 17 SDG according to the UN

2 Countries in the different clusters

We show explicitly the names of the countries belonging to each cluster in S1 Table 2.1. Notice that cluster Number -1 is not really a cluster, it groups dots classified by DBSCAN as not belonging to any cluster since they lie in low density zone. Nevertheless, we include them for completeness. Also notice that few countries are in more that one cluster. This means that during the time span of the analysis they changed their situation

The 10 countries which changed their classification: Bangladesh from '-1' (2000-2017) to cluster 4 (2018.2022); Benin from cluster 1 (2000-2017) to '-1' (2018-2022); Bulgaria from cluster 0 (2000-2006) to cluster 3 (2007-2022); Gabón from cluster 4 (2000-2001) to cluster 0 (2002-2022); Haiti from cluster 4 (2000-2020) to

Table 2.1. Countries grouped into clusters based on their SDG trajectories. The data underwent a sequential analysis: first, Principal Component Analysis (PCA) was utilized for dimensionality reduction. Then, t-Distributed Stochastic Neighbor Embedding (t-SNE) was applied to further map the data into a two-dimensional space, Finally, the DBSCAN clustering algorithm was used to group countries based on the resulting patterns.

Cluster	Countries
-1	Cyprus, United Arab Emirates, parts of the trajectories of: Bangladesh, Benin, Malta
0	Africa: Algeria, Cabo Verde, Egypt (Arab Rep.), Gabon, Morocco, Sao Tome and Principe, Senegal, Tunisia; America: El Salvador, Panama, Uruguay ; Asia: Georgia, Iran (Islamic Rep.), Jordan, Lebanon, Malaysia, Maldives, Russian Federation, Thailand, Türkiye; Europe: Albania, Bosnia and Herzegovina, Bulgaria, Montenegro; Oceania: Fiji
1	Africa: Angola, Benin, Cameroon, Congo (Dem. Rep.), Congo (Rep.), Cote d'Ivoire, Gambia (The), Guinea, Kenya, Liberia, Madagascar, Mozambique, Nigeria, Senegal, Sierra Leone, Tanzania, Togo; America: Haiti; Oceania: Papua New Guinea
2	America: Argentina, Brazil, Chile, Colombia, Costa Rica, Dominican Republic, Ecuador, Jamaica, Mexico, Peru, Uruguay, Venezuela (RB); Asia: Philippines
3	America: Canada; Europe: Belgium, Bulgaria, Croatia, Denmark, Estonia, Finland, Germany, Greece, Iceland, Ireland, Italy, Latvia, Lithuania, Malta, Netherlands, Norway, Poland, Romania, Slovenia, Sweden, United Kingdom; Oceania: Australia
4	Afria: Comoros, Djibouti, Gabon, Ghana, Mauritania, Mauritius, Namibia, Sao Tome and Principe, Somalia, South Africa, Sudan; America: Guatemala, Haiti, Honduras, Nicaragua; Asia: Bangladesh, China, India, Indonesia, Myanmar, Pakistan, Romania, Sri Lanka, Vietnam, Yemen (Rep.)
5	America: United States; Asia: Israel, Japan, Korea (Rep.); Europe: France, Portugal, Spain

cluster 1 (2021-2022); Malta from '-1' (2000-2010) to cluster 3 (2011-2022); Romania from cluster 4 (2000-2006) to cluster 3 (2007-2022); Sao Tome and Principe from cluster 4 (2000-2005) to cluster 0 (2006-2022); Senegal from cluster 1 (2000-2018) to cluster 0 (2019-2022); Uruguay from cluster 0 (2000-2004) to culster 2 (2005-2022).

3 Dynamics

In here we show the values of the coefficients of the second order equation used to fit the dynamical behavior of the goals. According to this the prediction of the dates to achieve the ideal score is also shown in S1 Table 3.1.

Cluster	a	b	c	year to zero
0	-2799.59	2.80247	-0.000700922	2048
1	-1881.52	1.89254	-0.000475281	2063
2	-669.863	0.686105	-0.000175162	2066
3	-269.743	0.279532	-0.000072022	2085
4	-2903.6	2.90572	-0.000726443	2054
5	-49.7872	0.0622445	-0.00001835	2101

Table 3.1. Best fitting coefficients of equation $r(t) = a + bt + ct^2$ for each cluster of countries obtained in S1 Table 2.1. The last column is the extrapolated time to plenty achieve all the goals. Fitting was done without 2020, 2021 and 2022 data, to suppress the effect of COVID pandemic.

4 The t-SNE dimensional reduction method.

The t-Distributed Stochastic Neighbor Embedding algorithm is a non-linear dimensionality reduction technique designed to visualize high-dimensional data by mapping it into a lower-dimensional space . Its main objective is to preserve the local structure of the data by ensuring that points that are close in the high-dimensional space remain close in the lower-dimensional representation.

4.1 Probability Distributions in High Dimensions

t-SNE starts by converting the pairwise Euclidean distances between points in the high-dimensional space into conditional probabilities. These probabilities represent the similarity between points. Specifically:

$$P_{j|i} = \frac{\exp\left(-\|x_i - x_j\|^2 / 2\sigma_i^2\right)}{\sum_{k \neq i} \exp\left(-\|x_i - x_k\|^2 / 2\sigma_i^2\right)}$$

Here, $P_{j|i}$ is the probability that point x_i picks x_j as its neighbor, assuming a Gaussian distribution centered at x_i with variance σ_i^2.

4.1.1 Perplexity

Perplexity is a crucial parameter in t-SNE that controls the balance between local and global aspects of the data structure. It can be interpreted as a smooth measure of the effective number of neighbors for each point. The perplexity is defined as:

$$\text{Perplexity}(P_i) = 2^{H(P_i)}$$

where $H(P_i)$ is the Shannon entropy of the conditional probability distribution P_i:

$$H(P_i) = -\sum_j P_{j|i} \log_2 P_{j|i}$$

Perplexity is typically chosen by the user and affects the value of σ_i for each data point, thereby controlling how the local neighborhood is defined.

4.2 Mapping to Lower Dimensions

In the lower-dimensional space, t-SNE aims to find a similar probability distribution Q_{ij} that reflects the similarities between points:

$$Q_{ij} = \frac{\left(1 + \|y_i - y_j\|^2\right)^{-1}}{\sum_{k \neq l} \left(1 + \|y_k - y_l\|^2\right)^{-1}}$$

Notice that in the lower-dimensional space, t-SNE uses a Student's t-distribution with one degree of freedom (essentially a Cauchy distribution) instead of a Gaussian distribution. This choice allows t-SNE to handle the so-called "crowding problem," ensuring that distant points are placed further apart.

4.3 Optimization Objective

The t-SNE algorithm seeks to minimize the Kullback-Leibler (KL) divergence between the probability distributions P_{ij} (in the original space) and Q_{ij} (in the lower-dimensional space):

$$KL(P\|Q) = \sum_{i \neq j} P_{ij} \log \frac{P_{ij}}{Q_{ij}}$$

The optimization adjusts the positions of the points in the lower-dimensional space to minimize this divergence, resulting in a configuration where similar points (high P_{ij}) are close together, and dissimilar points are far apart.
